# Development and validation of a prototype for the on-line simultaneous analysis of quality caprolactam synthesized on an industrial scale

**DOI:** 10.1016/j.mex.2022.101952

**Published:** 2022-12-05

**Authors:** Joaquín Hernández Fernández, David Rincón, Juan López-Martínez

**Affiliations:** a Chemistry Program, Department of Natural and Exact Sciences, San Pablo Campus, University of Cartagena, Cartagena 130015, Colombia; b Chemical Engineering Program, School of Engineering, Parque Industrial y Tecnológico Carlos Vélez Pombo Km 1 Vía Turbaco, Universidad Tecnológica de Bolivar, Cartagena 130001, Colombia; c Department of Natural and Exact Science, Universidad de la Costa, Barranquilla 080002, Colombia; d Centro de investigación e invención en ciencias e ingeniería, CECOPAT & A, Cartagena, Colombia; e Institute of Materials Technology (ITM), Universitat Politecnica de Valencia (UPV), Plaza Ferrandiz ‘ and Carbonell s/n, Alcoy, Alicante 03801, Spain

**Keywords:** Caprolactam, Quality assurance, Prototype, Rapid method for the determination of impurities in caprolactam in situ

## Abstract

Caprolactam is a highly useful monomer obtained through the Beckmann arrangement, which generates large profits worldwide and is widely used in different industries. During the synthesis process, various components can be generated that weaken the quality of the final product, to have control of the monomer, monitoring is carried out during the synthesis and characterization of the final product. These characterizations generally take time due to the different techniques that must be performed to obtain the data. In this work, a method is designed that associates different techniques to reduce the number of steps carried out in the tests to determine the quality of the material, optimize the times and generate a quality and efficient process in a shorter time, in addition, it is due to a semi-automated system for the simultaneous characterization of caprolactam, which, according to the statistical data obtained for sodium, iron, volatile bases, and moisture analysis were reproducible. The developed prototype had 21 on-line valves that allowed taking the representative volumes of samples and reagents necessary for each measurement. There is excellent linearity where the correlation coefficient has values between 0,9992 and 1. The values obtained for the relative error are between 0.18 and 2.24% for laboratory tests using the traditional method and between 0.21 and 3.83% for tests carried out using the prototype. The P value of the evaluation of the means was 0.997, indicating that the means are not statistically different.

• Caprolactam analysis
• Process optimization
• Determination of impurities

## Graphical abstract

**Figure:**
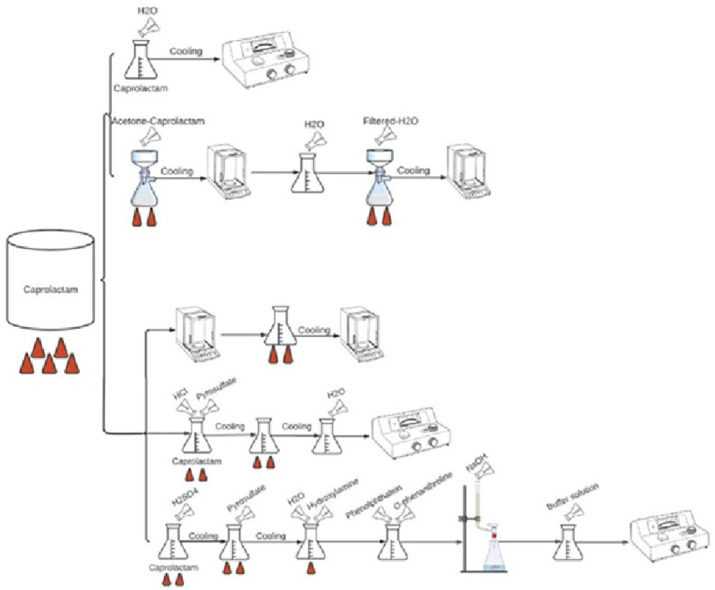

Specifications table

**Table:** 

Subject Area:	Chemistry
More specific subject area:	Analytic chemistry
Method name:	Rapid method for the determination of impurities in caprolactam in situ
Name and reference of original method:	DSM 379 E, DSM 100 −1E, NTC 1206
Resource availability:	N.A.

## Introduction

**Fig. 1:**
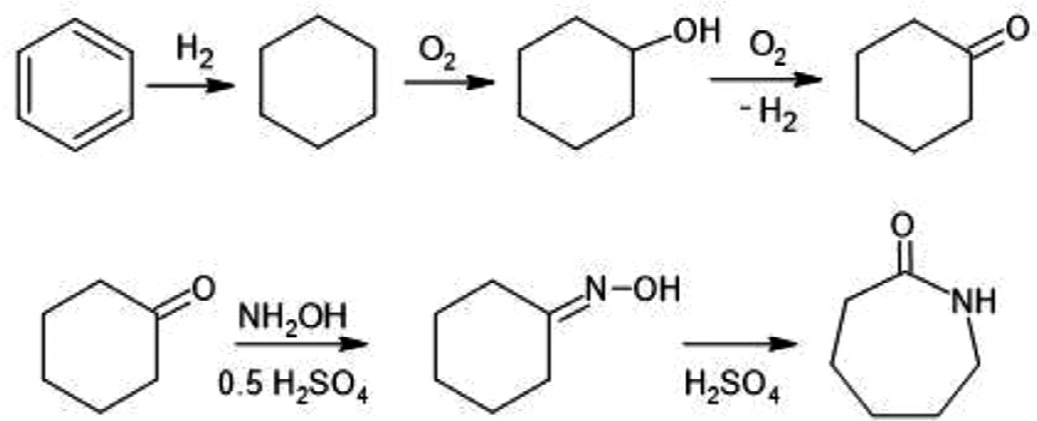
Beckmann rearrangement.

Caprolactam is an organic compound belonging to cyclic amides or lactams with the formula C_6_H_11_NO, widely used in different industries such as the automotive, textile, and electronic industries and, the production of polyamides such as Nylon 6, among others [1,2]. Caprolactam can be obtained using different pathways of strains that use toluene, benzene, and cyclohexane that react by Beckmann rearrangement and the formation of cyclohexanone as an intermediate [3], [4], [5], [6]. This process consists of the oxidation of cyclohexane to cyclohexanone followed by oxylation to obtain as an intermediate the cyclohexanone-oxime, which passes to a rearrangement process becoming caprolactam [6], [7], [8], as you can see in Fig. 1.

The caprolactam market reported by 2021 a global market size of USD 12.3 trillion with a great capacity for growth, with the Asia Pacific dominating this market [9]. Moisture contents greater than 0.2% affect the size of the macromolecule and have an impact on the glass transition temperature of the polymer it forms [10], [11], [12], extinction with levels greater than 0.1 show a low purity in the synthesized caprolactam and it has been established that the higher the excitation values, the lower the concentration of caprolactam [13]. Impurities denoted as insoluble material with levels greater than 10 ppm generate a decrease in the polymerization efficiency of Nylon 6 [13]. the sodium content affects the pipes and equipment through which caprolactam is transported, so it is recommended that it be in concentrations below 250 ppm, causing corrosion. And the ash content with concentrations above 10 ppm.

**Fig. 2:**
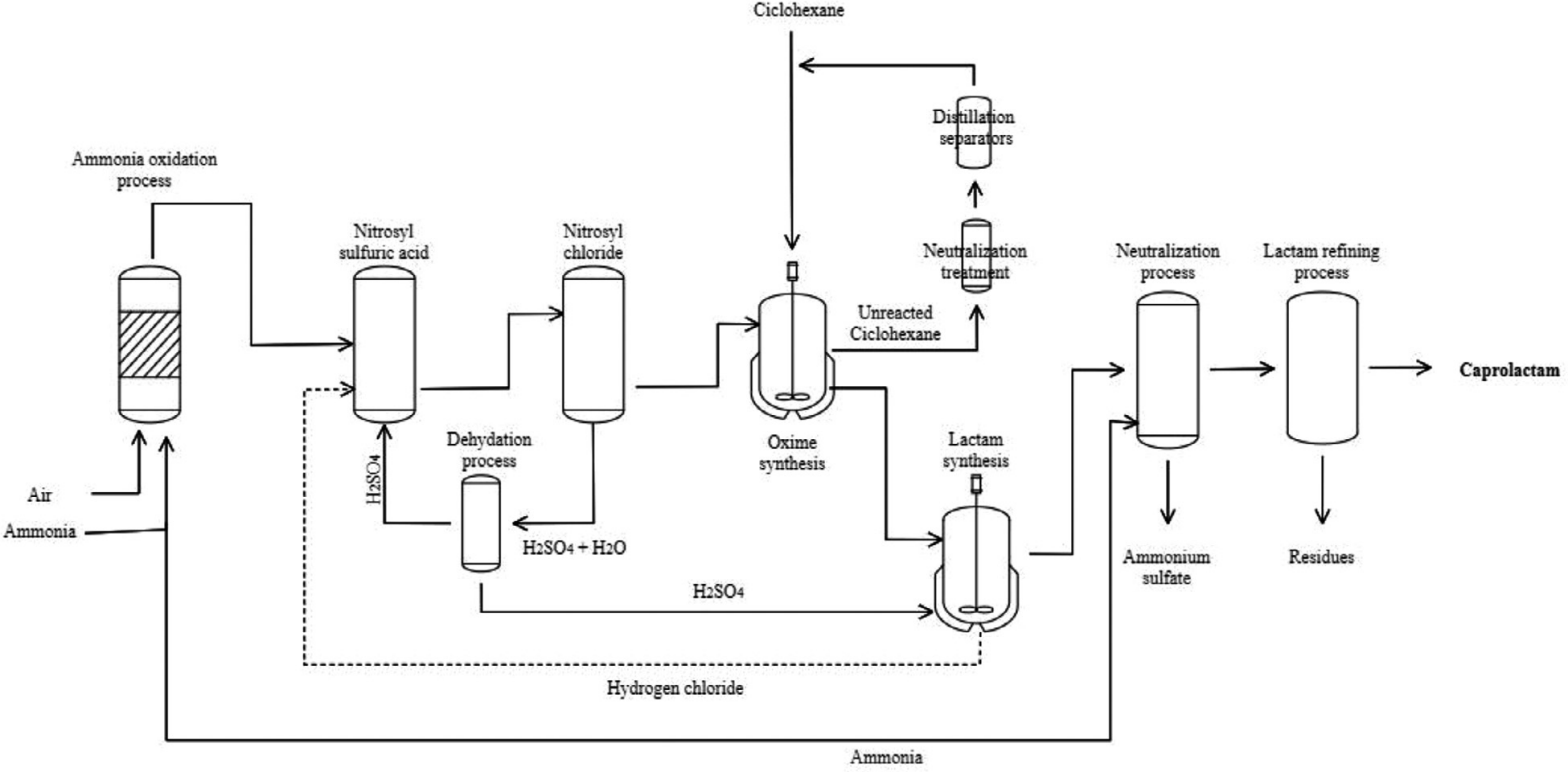
Scheme of caprolactam production.

Given the negative effect of these impurities on the production process, on the quality of caprolactam, the quality of Nylon 6, and the different applications of these materials, especially in the textile sector, is the reason why a new online measurement system for these impurities is proposed around the world. Petrochemical plants producing this monomer at the international level use classical and sometimes instrumental analytical methodologies to independently quantify these impurities. Automated and fast measurements are of vital interest as they allow plant operators, statistical process control areas, and engineering departments to take quick actions to optimize the process in such a way that it guarantees the implementation of preventive measures and some scenarios corrective measures to reduce the impurities present in the product. In process or in each of the different stages that are part of the production process on an industrial scale, to increase the efficiency of the process. During the synthesis of this material, different stages are presented where affectations can be generated that lead to caprolactam not complying with the established quality standards. Fig. 2 shows the industrial-scale process of caprolactam production [14]. This process consists of the following 4 stages: 1. Formation of an ammonium salt. 2. Oxidation of acetone with the ammonium salt obtained. 3. Beckmann rearrangement, together with neutralization with ammonia, and 4. Purification of the caprolactam solution obtained [7,8].

At the international level, the evaluations of the content of impurities in the stages of interest of the synthesis process are carried out by the NTC 1206 standard [15], where a series of individual laboratory tests that determine the viability of being used for the textile industry and the ranges of acceptable values that caprolactam must meet are shown, which are shown in Table 1. These tests are manual, both in the sampling at the different sampling points and in the different tests carried out inside the laboratory. These manipulations of the samples are characterized by being susceptible to external manipulations and with high time consumption for the execution of the analytical tests. Due to the good results obtained in the systematization and simultaneity of chromatography analysis through the implementation of 2, 4 and 6-way valves [16], [17], [18], this same principle is applied for the adaptation of laboratory analysis to scale them to caprolactam producing plants. Therefore, this prototype design proposes a semi-automation of these tests that allow simultaneous performance and automatic calculation for the reduction of laboratory time.

**Table 1:** Maximum allowable value for caprolactam in each test.

Test	Unit	Max
Moisture	% weight	0,20
Ash	ppm	10
Iron	ppm	1
Insoluble material	ppm	10

## Methods

### Scheme of classic analyses in the laboratory

**Fig. 3:**
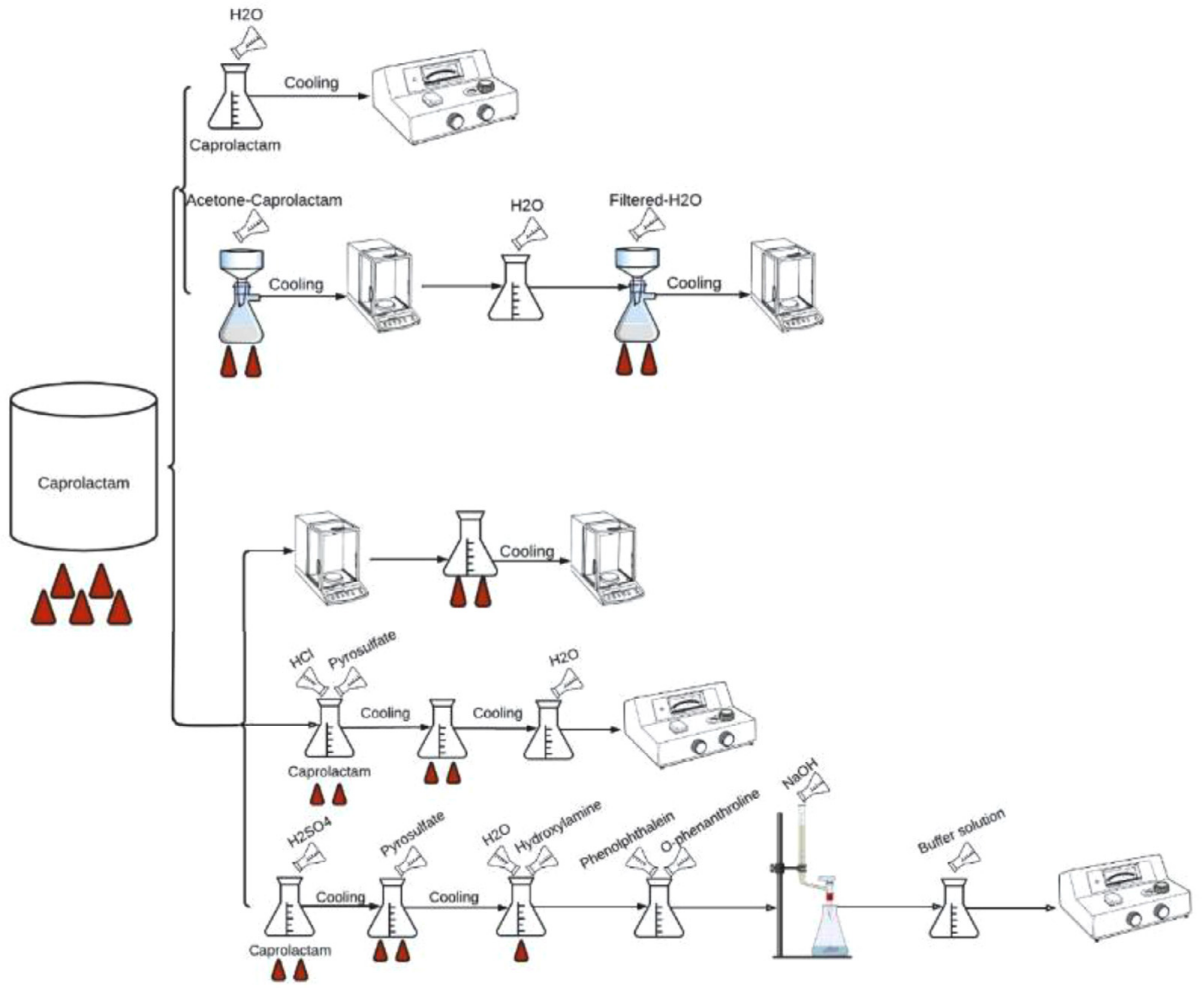
General scheme of laboratory-scale analysis.

Fig. 3 shows the different analyses performed on caprolactam to determine its quality. It starts by taking a portion of caprolactam and heats up until the sample melts. The calcined sample is divided into two, the first is left to cool in the solid-state and the second is dissolved and then left to cool. The previous portion is divided to perform two procedures where in one an aliquot of 50 gr is taken and dissolved in 50 ml of water, carrying out the determination of the extinction of the caprolactam, using a spectrophotometer at a wavelength of 290 nm, and Eq. (1) is solved. In the other 100 ml of acetone is used to dissolve the caprolactam, filtered and left to dry for 1 h at 110 °C for further cooling and weighing (gr0), then 250 gr of the sample is dissolved in 300 ml of water, filtered again and dried in 30 min at 110 °C, it is left to cool, and the final weight (gr1) is taken for the determination of insoluble by Eq. (2).

(1) $$\frac{Absorbance\;to\;290nm\;x\;50}{b}=Extinction\;\left(290\;nm\right)$$

(2) $$\frac{\left(g_{1}-g_{0}\right)*10^{6}}{W\;\left(gr\right)}=ppm\;insoluble$$

The calcined sample that was left cooling and subdivided into three portions, one of them is taken and mixed with hydrochloric acid and pyrosulfate to perform a subsequent heating, then it is left to cool and 1 ml of H_2_SO_4_ is added to again carry out heating where the SO_3_ gases will be removed present in the solution; the sample is then melted at 800 °C for 10 min and left to cool; the calcined portion is placed in a beaker and water is added until the 100 ml is completed and a standard solution with 1 ml of H_2_SO_4_ is established for a subsequent zero calibration of the spectrophotometer and find the sodium content in the caprolactam sample determined from the data offered by the team and Eq. (3).

(3) $$\frac{Lect\;-\;a}{b}\times \frac{100}{\;W\left(g\right)}=ppm\;Na$$

Another section of the calcined sample is taken that was left cooling and mixed with H_2_SO_4_ to perform a heating where the SO_3_ gases present in the solution are removed, then the sample is melted at 800 °C for a time of 10 min and left cooling, then 3 gs of pyrosulfate are added for a last casting and cooling process. The calcined sample is added 5 ml of hydroxylamine and 15 ml of water, gently heated 5 min, cooled, and added 5 ml of Ortho-Phenanthroline and 3 drops of phenolphthalein to start a titration with NaOH 4 N until the solution turns to pink, after 10 min 10 ml of buffer solution pH 4 is added and left to stand for 30 min, simultaneously, calibrate the spectrophotometer to a wavelength of 510 nm for when the sample has rested to obtain the absorbance and the amount of iron in the caprolactam using Eqs. (4) and (5), where a and b depend on the spectrophotometer in which it works

(4) $$\frac{Abs\;-a}{b}=mg\;Fe$$

(5) $$\frac{mg\;Fe\;*1000}{W}=ppm\;Fe$$

The third portion that was left cooling is taken and the sample is weighed (gr0), the test is continued by melting the amount obtained in a fractional way for 40 min at 800 °C and a cooling is carried out in a desiccant, to again weigh the amounts (gr1) and with this data Eq. (6) is used to find the ashes.

(6) $$\frac{\left(g_{1}-g_{0}\right)*10^{6}}{W\;\left(gr\right)}=ppm\;Ash\;$$

Finally, a caprolactam sample is taken and mixed with 30 ml of methanol and Karl Fischer solution to bring to anhydrous state, then the equipment is standardized to 10 mmA at least 10 s for use. 10 gr of the sample are taken in a beaker and the Karl Fischer reagent is added until the same end point is found and the amount of reagent that was consumed is read to find the Karl Fischer reagent factor, 2 or 3 drops of water are added (determine how many ml H_2_O were used) and finally the Karl Fischer reagent is added again to obtain of the endpoint in ml (10 milliamps for at least 10 s). The data obtained in the procedure are used to enter them into Eq. (7) and find the moisture content.

Karl Fischer reagent factor

(7) $$\begin{array}{ccc} & & \left(W_{1}-W_{0}\right)\;*1000=mg\;of\;water\\ & & \frac{mg\;\text{of}\;water}{V\;ml}=F\\ & & \frac{\left(V*F*100\right)}{\left(W*100\right)}=\%\;H_{2}O \end{array}$$

### Prototype of the systematization of the simultaneous analysis

**Fig. 4:**
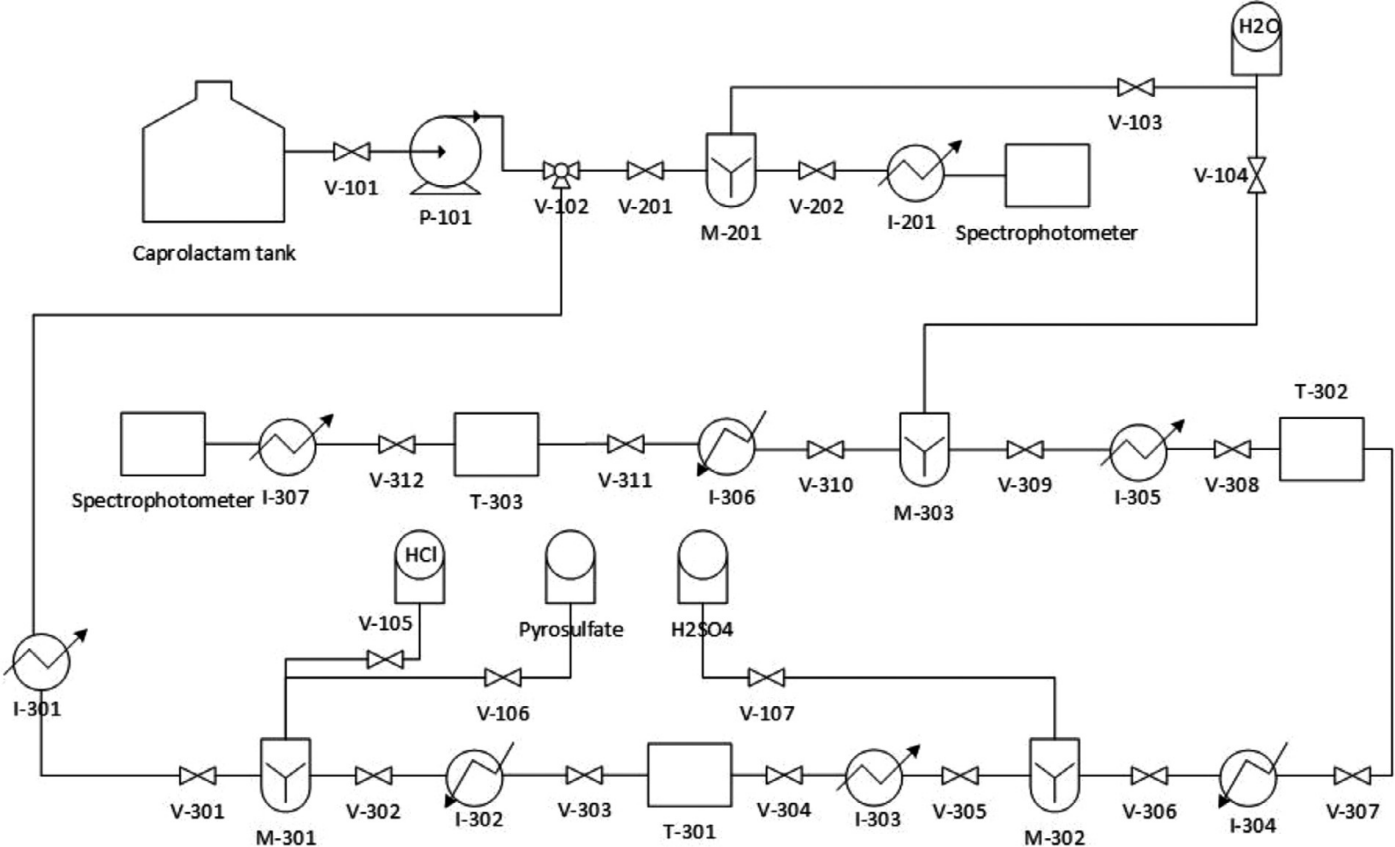
General prototype scheme of the simultaneous analysis.

Using the procedure established at the laboratory scale, a prototype is designed for the systematization of the analysis by means of the semi-automation of valves as shown in Fig. 4 and the table of valve events is shown in Table 2.

**Table 2:** Table of valve events of the simultaneous analysis prototype.

Valve	Time on (min)	Comments
V-101	0,01	Caprolactam passage
V-102	0,01	Caprolactam passage
V-103	1	Water passage
V-104	35,2	Water passage
V-105	2,3	HCl Step
V-106	3,1	Pyrosulfate passage
V-107	30,5	H2SO4 Step
V-201	0,5	Caprolactam passage
V-202	5,2	Mixture
V-301	0,5	Caprolactam passage
V-302	8,2	Mixture
V-303	8,5	Mixture
V-304	10,2	Mixture
V-305	30,2	Mixture
V-306	32,5	Mixture
V-307	32,5	Mixture
V-308	35	Mixture
V-309	35	Mixture
V-310	40,5	Mixture
V-311	60,5	Mixture
V-312	80	Mixture

The valve V-101 and V-102 are open to allow the passage of caprolactam through the pipe that carries the flow through valves V-201 and V-301 while the others are closed, where simultaneous procedures are presented, where the first allows the opening of the V-103 and subsequent closure to obtain a caprolactam-H_2_O solution and when this mixture has occurred, the V-202 is opened for its passage through the spectrophotometer and find the extinction, resuming with the V-301 valve, the flow of caprolactam and subsequent closure is allowed, while the rest of the valves are closed so that the valves V-105, V-106 have an opening and subsequent closing, where a mixture of caprolactam, HCl and pyrosulfate was witnessed. Then, the V-302 opens so that the mixture can pass through a heater and the removal of SO_3_ vapors is given, the V-303 valve allows the hot fluid to be stored and eventually pass through the V-304 to cool the mixture.

The V-305 valve opens allowing the passage of the cooled fluid, while the V-107 also gives opening for obtaining a solution with H_2_SO_4_, the V-306 is activated to perform a heating process and the V-307, to store the obtained solution. The V-308 is actuated to allow the passage of the fluid for a cooling process, the V-309 valve is actuated and then the V-104 so that the solution that is transported through the tubes is dissolved in water, the V-310 is activated so that the solution goes through a heating process and the V-311 to store the solution that was heated, the V-312 is operated for a subsequent cooling of the solution and this passes to the spectrophotometer.

## Method validation

Considering the proposed model where a prototype was designed that executes the different tests simultaneously, we proceeded to characterize a sample of caprolactam from the same batch under the same operations to evaluate the content of iron, ash, material insoluble in water and the extinction present [17].

### Linearity

The concentration of the impurities was found to be linear. The quality of the findings is determined by the laboratory's and prototype's operation and functionality; therefore, its operation is confirmed based on its linearity. For each of the compounds of interest, four concentration levels were utilized to determine linearity. The iron concentrations were 0.35, 0.6, 1, and 1.5 ppm. moisture levels of 0.1, 0.2, 0.3, and 0.5%, sodium concentrations of 150, 200, 250, and 300 ppm. Insoluble concentrations of 3.0, 5.0, 10, and 15 ppm. Ash content of 3.0, 5.0, 10, and 15 ppm. Extinction 0.01, 0.03, 0.05, and 0.08 nm, respectively. Linearity was assessed visually as response versus impurity concentrations and given as the correlation coefficient (r^2^) [17], [18], [19].

The data obtained are measured in Table 3, demonstrating that regardless of the test being performed or the impurity that is being evaluated, there is excellent linearity where the correlation coefficient (r) has values between 0,9992 and 1.

**Table 3:** Determination of linearity.

Test	Unit	Theoretical value	Laboratory value	Prototype value	Equation	r^2^
Iron	ppm	0,3	0,3	0,3	*y* = 1.002x - 0.0017	0,9992
		0,6	0,6	0,6		
		1	1	1		
		1,5	1,5	1,5		

Ashes	ppm	3	3	3	*y* = 1.0075x - 0.1118	0,9997
		5	5	5		
		10	10	10		
		15	15	15		

Insoluble material	ppm	3	3	3	*y* = 1.001x - 0.0031	1
		5	5	5		
		10	10	10		
		15	15	15		

Extinction at 290 nm	nm	0,01	0,01	0,01	*y* = 1.0123x - 0.00008	0,9999
		0,03	0,03	0,03		
		0,05	0,05	0,05		
		0,1	0,1	0,1		

Moisture	%	0,1	0,1	0,1	*y* = 0.9994x + 0.0007	1
		0,2	0,2	0,2		
		0,3	0,3	0,3		
		0,5	0,5	0,5		

Sodium	ppm	150	150	151	*y* = 0.999x +1.1	1
		200	201	201		
		250	250	250		
		300	300	301		

### Precision and accuracy

Precision was measured in terms of repeatability (intraday precision), represented as a relative standard deviation, and assessed at three concentration levels: low, medium, and high. Six repetitions of three concentration levels were used to achieve repeatability for the amount of iron, moisture, sodium, insoluble, ashes, and extinction. Each laboratory and prototype operation was carried out on the same day by the same operator using the same instrument.. If the average overall values were less than 20%, the within-day accuracy was considered verified [20,21]. As an acceptability criteria, a divergence of less than 15% of the predicted value is proposed [18,^20^,21]. The relative errors (Er) were used to make the computations. Precision was deemed ideal when the bias was less than 15% and acceptable when it was between 15% and 20%. Following the selection of the samples, the enhanced samples were prepared. The values obtained for the calculation of precision and accuracy are shown in Table 4. Then the performance of the prototype was evaluated by comparing its pressure and accuracy with the results obtained in a classical way with the laboratory method. The measurement ranges for the analysis of iron, ash, insoluble material, extinction at 290 nm, humidity and sodium were 0.3–0.6 ppm, 3–10 ppm, 3–10 ppm, 0.01–0.05 nm, 0.1–0.3% and 200–300 ppm respectively. The performance of the two measurement systems is monitored analyte by analyte, in order to identify their punctual error and then identify specific improvement opportunities. The comparisons of the RSD for laboratory Vs Prototype in the iron content were (0.8–2.6 Vs 0.9–3.2), Ashes (0.2–1.2 Vs 0.4–1), insoluble material (0.2–1.7 Vs 0.2–1.2), extinction 290 nm (0.7–2.2 Vs 1.7–5), Moisture (0.3–2.2 Vs 0.5–1.5) and Sodium (0.1–0.2 Vs 0.1–0.3) respectively. All RSD values ​​for each of the measurement variables of interest were lower than the 15% value that the literature considers acceptable for intermediate precision. The comparisons of Er for laboratory Vs Prototype in the iron content were (0.6–2.2 Vs 0.7–1.9), Ashes (0.2–0.9 Vs 0.3–0.8), insoluble material (0.1–1.3 Vs 0.2–0.8), extinction 290 nm (0.7–1.9 Vs 1.2–3.8), Moisture (0.3–1.5 Vs 0.4–1.3) and Sodium (0.2 Vs 0.2) respectively. All the Er were lower than the value of 15% that the literature considers as acceptable for Accuracy.

**Table 4:** Precision and accuracy in laboratory and prototype results.

Practice	Unit	Theoretical value	Laboratory			Prototype		
			*Value found^a^ ± s*	RSD (%)	Er (%)	*Value found^a^ ± s*	RSD (%)	Er (%)
Iron	ppm	0,3	0.3 ± 0.003	0,8	0,6	0.3 ± 0.005	1,5	1,
		0,6	0.6 ± 0.005	0,85	0,65	0.6 ± 0.005	0,9	0,7
		1	1. ± 0.026	2,6	2,2	1 ± 0.032	3,2	1,9

Ashes	ppm	3	3.0 ± 0.038	1,2	0,9	3.01 ± 0.03	1,0	0,8
		5	4.8 ± 0.034	0,7	0,5	4.75 ± 0.04	0,9	0,7
		10	10 ± 0.022	0,2	0,2	10.01 ± 0.038	0,4	0,3

Insoluble material	ppm	3	3.0 ± 0.052	1,7	1,3	3.0 ± 0.03	1,2	0,8
		5	5 ± 0.0242	0,5	0,4	5 ± 0.03	0,7	0,4
		10	10 ± 0.021	0,2	0,1	10.01 ± 0.02	0,2	0,2

Extinction 290 nm	nm	0,01	0.01 ± 0.0002	2	1,9	0.01 ± 0.0005	5,0	3,8
		0,03	0.03 ± 0.0002	0,7	0,7	0.03 ± 0.0005	1,7	1,2
		0,05	0.05 ± 0.001	2,2	1,8	0.05 ± 0.002	4,1	2,8

Moisture	%	0,1	0.1 ± 0.022	2,2	1,5	0.1 ± 0.0005	0,5	0,4
		0,2	0.2 ± 0.008	0,8	0,6	0.2 ± 0.003	1,5	1,3
		0,3	0.3 ± 0.001	0,3	0,3	0.3 ± 0.003	0,9	0,8

Sodium	ppm	200	201 ± 0.503	0,2	0,2	201 ± 0,53	0,3	0,2
		250	250 ± 0.421	0,2	0.2	250 ± 0.46	0,2	0,2
		300	300 ± 0.401	0,1	0.2	301 ± 0.45	0,1	0.2

S, standard deviation; RSD, relative standard deviation; Er, relative error. ^a^ Calculated value of the standard calibration curve for six determinations on the same day.

**Fig. 5:**
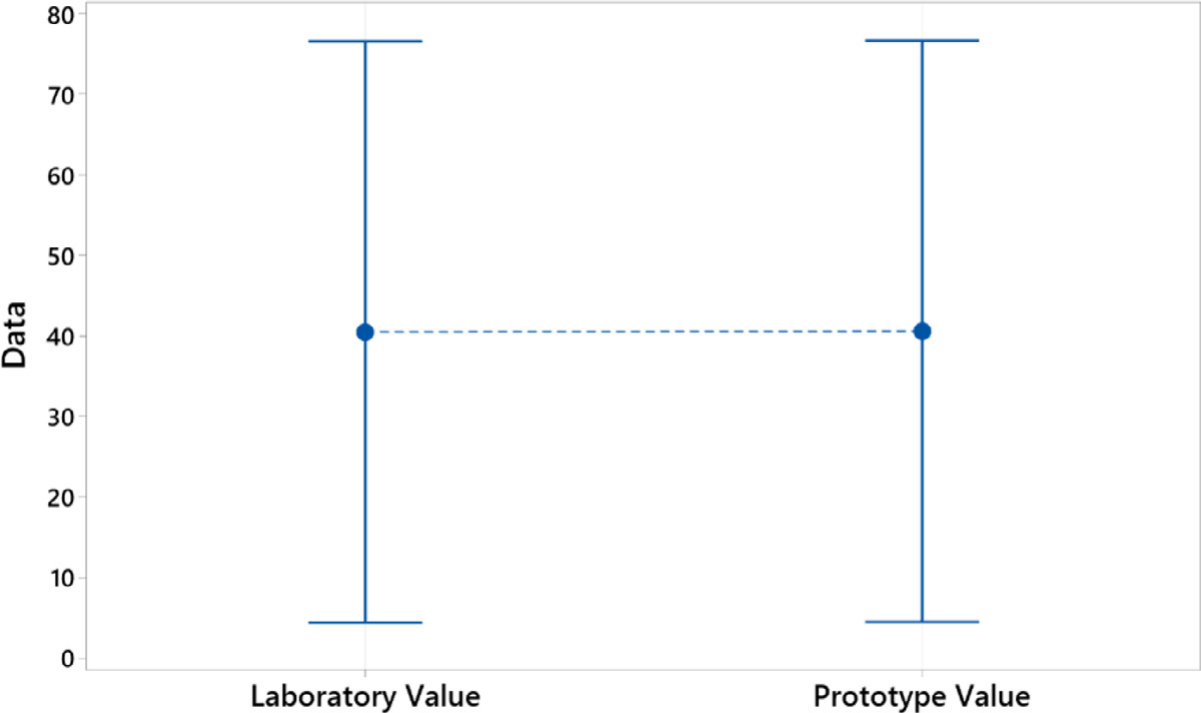
95% confidence interval for the mean laboratory and prototype.

**Fig. 6:**
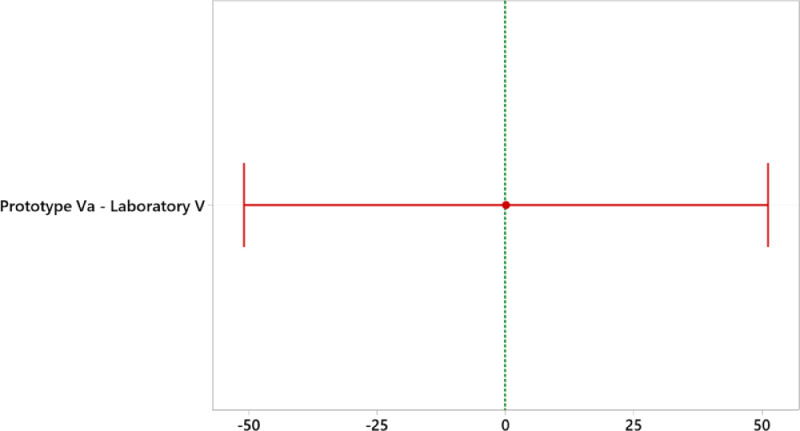
Tukey simultaneous 95% Cis. Difference of means for laboratory and prototype.

In this investigation we use ANOVA to determine if any of the differences between the means are statistically significant and for this we compare the p-value with the level of significance to evaluate the null hypothesis. The null hypothesis tells us that the population means of the data are all equal. Typically, a significance level (denoted as α or alpha) of 0.05 works well. A significance level of 0.05 will indicate a 5% risk of concluding that there is a difference when there is no real difference. For p-value ≤ α, the differences between some of the means are statistically significant. For p-value > α, the differences between the means are not statistically significant. So in Table 5 and Fig. 5, we present the one-way ANOVA for the laboratory tests and the tests of the proposed prototype. The results clearly show us that the p value was 0.997, a value that is greater than the significance level of 0.05, indicating that our proposed research does not have enough evidence to reject the hypothesis that the population means are all the same. Therefore, the experiments carried out around the proposed prototype are accepted as adequate and analytically reliable to replace the laboratory technique in any situation in the measurement of iron, ashes, insoluble materials, extinction at 290 nm, humidity and sodium content in the samples. of caprolactam. We have also applied Tukey's method in ANOVA. See Table 5 Fig. 6. With Tukey we create confidence intervals for all pairwise differences between factor level means while controlling for the error rate per family at a specified level. It is important to consider the family error rate when making multiple comparisons, because the probability of making a type I error for a series of comparisons is greater than the error rate for any single comparison. To counteract this higher error rate, Tukey's method adjusts the confidence level of each individual interval so that the resulting simultaneous confidence level is equal to the defined value. Table 5 shows that the letter A is shared between the laboratory values ​​and the prototype, thus demonstrating their equality. In addition, Fig. 6 shows that the interval of interest continues at zero, indicating that they are not statistically different.

**Table 5:** One-way ANOVA: laboratory value; prototype value.

Analysis of Variance							
Source	DF	Seq SS	Contribution	Adj SS	Adj MS	F-Value	P-Value
Factor	1	0		0%	0	0.08	0.997
Error	46	354,304		100%	354,304	7702.27	
Total	47	354,304		100%			
Means							

Factor	N	Mean		StDev		95% CI	

Laboratory Value	24	40.5		87.7		(4.4; 76.5)	
Prototype Value	24	40.6		87.9		(4.5; 76.6)	
Tukey Method and 95% Confidence							

Factor	N	Mean		Grouping		Adjusted P-Value	

Laboratory Value	24	40.5		A		0.997	
Prototype Value	24	40.6		A

## Conclusions

This procedure it is possible to semi-automate part of the tests carried out to determine the quality of the caprolactam at an industrial level, reducing the analysis time, which allows adjustments to be made in the process controls immediately to avoid that more products can be generated without complying with all the quality parameters. The proposed model represents an optimal alternative for the semi-automation of the characterizations made to the caprolactam samples in order not only to carry out a systemic and simultaneous process, but also one that allows a reduction in operating times and is of benefit to the last processes in the production of caprolactam and can potentially be used in other stages of synthesis to avoid large-scale production of out-of-specification caprolactam.

## Declaration of Competing Interest

The authors declare that they have no known competing financial interests or personal relationships that could have appeared to influence the work reported in this paper.

## Data Availability

I have shared my data in the article.
